# Recent Advances in Vanadate-Based Materials for Photocatalytic Hydrogen Production

**DOI:** 10.3390/molecules30040789

**Published:** 2025-02-08

**Authors:** Kandasamy Sasikumar, Heongkyu Ju

**Affiliations:** Department of Physics, Gachon University, Seongnam-si 13120, Gyeonggi-do, Republic of Korea; sasiphy2022@gachon.ac.kr

**Keywords:** H_2_ production, heterojunctions, photocatalysis, synthesis, vanadates, water splitting

## Abstract

Metal vanadates are a developing group of semiconducting metal oxide materials that are gaining increasing attention due to their great redox potential, effective separation of photogenerated electron–hole pairs, and tunability of structural and physicochemical properties. Their rational design as effective photocatalysts can find use in various applications, including energy conversion/storage and environmental remediation. In particular, one of the viable ways to address energy-related issues can be through the sustainable production of hydrogen (H_2_), a clean fuel produced by photocatalysis using metal vanadates. However, the rapid recombination of photogenerated electron–hole pairs limits their practical use as effective photocatalysts, and thus, many efforts have been devoted to optimizing metal vanadates to enhance their efficiency. Herein, we provide a comprehensive review that deals with the recent development strategies of metal (Ni, Fe, Zn, Ag, In, Bi, rare earth, etc.) vanadates with the working mechanisms. Their synthesis, doping, cocatalyst loading, heterojunction creation, and carbon loading are also reviewed for photocatalytic H_2_ production. The challenges that metal vanadate-based photocatalysts have been facing are also discussed along with their significant potential for environmentally friendly and sustainable clean fuel production.

## 1. Introduction

Growing concerns over environmental pollution and depletion of natural resources for energy harvest are urging researchers to explore alternative, renewable, and clean energy sources for the future [1,2,3,4]. Hydrogen (H_2_), one of the emerging energy vectors, is gaining attraction due to its clean transition/storage of energy. Photocatalytic H_2_ production has been considered a promising approach for direct solar energy-to-H_2_ conversion. H_2_ is one of the most sustainable carbon-free energy vectors and has become a highly preferred alternative to a next-generation fuel due to its properties, such as high energy yield (122 kJ g^–1^), sustainability over the life cycle, renewability, environmental friendliness, and cost-effective handling [5,6].

**Figure 1:**
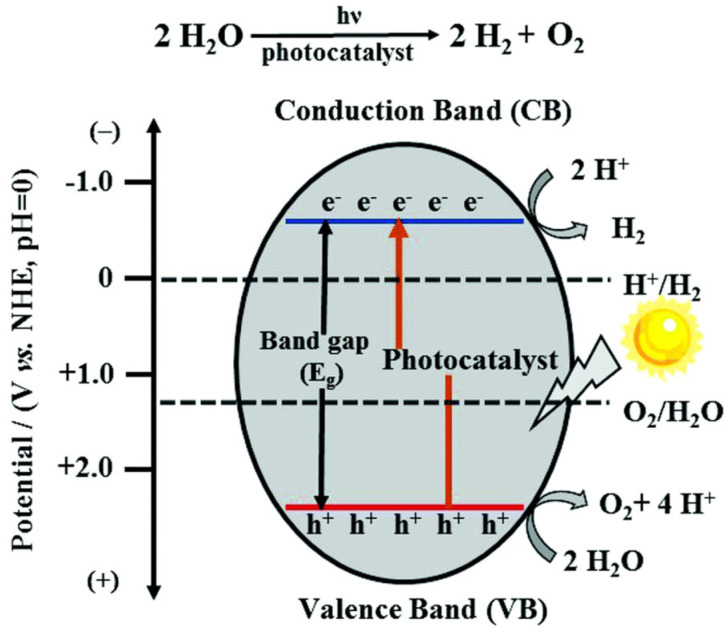
Schematic of water splitting on the surface of semiconductor photocatalyst [8]. Copyright 2019, Royal Society of Chemistry.

Photocatalytic H_2_ production through overall water (H_2_O)-splitting technology is defined as the splitting of H_2_O molecules into H_2_ and O_2_ over a semiconductor material. The process typically involves the following steps. First, photons are absorbed by the semiconductor photocatalyst, where electrons are excited from the valence band (VB) to the conduction band (CB) to create electron–hole pairs; second, the photoexcited electron-hole pairs are separated and migrated to the semiconductor surface; third, the protons of hydrogens of H_2_O accept photogenerated electrons, generating H_2_ by reduction, whereas free holes combine with electrons of oxygen ions of H_2_O to produce O_2_ by oxidation. More specifically, for photocatalytic reduction (2H_2_O + 2e^−^ → H_2_ + 2OH^−^ at pH 0), the CB edge should be above the H^+^/H_2_ energy level associated with the reduction potential (0 V with respect to the normal hydrogen electrode (NHE) at pH 0). Meanwhile, for photocatalytic oxidation (2H_2_O + 2h^+^ → O_2_ + 4H^+^), the VB edge should be below the O_2_/H_2_O energy level associated with the oxidation potential (1.23 V with respect to the NHE at pH 0). This follows that the desirable band gap (E_g_) of the semiconductor photocatalyst should be greater than 1.23 eV (Figure 1). Thus, the energy levels of CB and VB edges are key to determining its photocatalytic performance, although other factors, such as reaction kinetics and its thermodynamic stability, influence the reaction efficiency [7,8,9]. However, even if such requirements of energy band levels are satisfied, the semiconductor photocatalyst would still face challenges that limit reaction efficiency. These challenges include ineffective light absorption, the inapt energy band structure, and the non-negligibly high rate of recombination of photogenerated electron–hole pairs (non-radiative and radiative decays of the photogenerated carriers) [9]. To overcome the aforementioned problems, various material systems have been investigated, including those based on oxides [10,11,12], sulfides [13,14], phosphides [15], carbon materials [16,17], metal–organic frameworks (MOFs) [18], covalent organic frameworks (COFs) [19], and Mxene nanostructures [20].

Vanadates have garnered significant attention in pollutant degradation and water splitting due to their efficiency in harvesting visible light energy, high chemical stability, and substantial catalytic activity [21,22,23]. Metal vanadates, including MVO_4_, MV_2_O_4_, MV_2_O_6_, MV_3_O_8_, M_2_V_2_O_7_, and M_3_V_2_O_8_ (where M = alkaline earth/transition/rare earth/other metals), are considered to be effective for photocatalytic H_2_ production. Vanadium permits multiple oxidation states (V^2+^, V^3+^, V^4+^, and V^5+^), facilitating effective redox reactions with high stability. For instance, rare earth-based vanadates were reported for purification of water and air, H_2_/O_2_ production, photoreduction of CO_2_, oxidative desulfurization, and biomass conversion [24]. BiVO_4_, InVO_4_, and Zn_3_V_2_O_8_ were developed for pollutant degradation, CO_2_ reduction, H_2_ evolution, and N_2_ fixation applications [25]. Very recently, vanadate-based materials including (Ca, Sr, Ba)V_2_O_6_, Ce-BiVO_4_/SrTiO_3_ Z-scheme heterojunction, and Ni-doped PbS quantum dots on WO_3_/BiVO_4_ have been reported as effective photocatalysts for H_2_ production [22,26,27].

In this review, we particularly focus on vanadate-based materials for H_2_ production not just because of the fact that few review papers are available on this subject but because of their importance as the new material system for green energy harvest. The recent development of vanadates for photocatalytic H_2_ production is systematically covered with the following steps. First, we introduce the mechanisms of different heterojunctions built from various vanadate systems. Next, an overview of synthesis techniques for vanadates is briefly presented. The various vanadate-based photocatalysts to be dealt with are in the form of nanocomposites, heterojunctions, and solid solutions. We also discuss various physicochemical factors affecting their photocatalytic performance. Lastly, a concise summary of the key challenges and future opportunities with the vanadate material system is presented.

## 2. Heterojunctions—Types and Mechanisms

**Figure 2:**
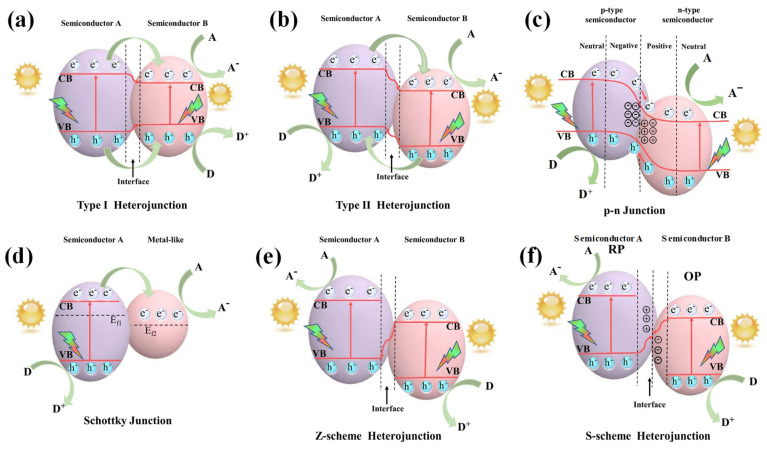
Band structures of (**a**) Type-Ⅰ, (**b**) Type-Ⅱ, (**c**) p–n, (**d**) Schottky, (**e**) Z-scheme, and (**f**) S-scheme heterojunctions (A: electron acceptor; D: electron donor; E_f_: Fermi level; CB: conduction band; VB: valence band; RP: reduction photocatalyst; OP: oxidation photocatalyst) [30]. Copyright 2024, Elsevier.

Constructing heterojunctions is an efficient strategy to extend the light absorption range and improve the separation of photoexcited charge carriers [28,29,30]. Therefore, we attempt to classify heterojunctions according to their component properties, carrier transfer mechanism, and energy band structure, as seen in Figure 2 [31]. The heterojunction can be defined as an interfacial region formed between semiconductor-A (SC-A) and semiconductor-B (SC-B) with different band structures, by which an interfacial potential difference is developed, leading to the creation of a built-in electric field. This internal electric field (IEF) influences the electrons and holes to move in the opposite direction, which leads to charge separation as electrons are migrated to the CB with a high reduction potential, whereas the holes are migrated to the VB with a high oxidation potential. As a result, the heterojunction’s charge carrier dynamics boost photocatalytic performance by promoting effective charge separation, rapid charge transfer, and improved lifespan of electron–hole pairs. The conventional heterojunction structures can be categorized as Type-I (straddling gap), Type-II (staggered gap), and Type-III (broken gap). In addition, due to the recent developments in photocatalytic technology, p–n heterojunction, Schottky junction, Z-scheme, and S-scheme heterojunction have also received more attention from researchers [32,33,34]. To date, several vanadate-based heterojunctions have been developed in the field of photocatalytic H_2_ evolution.

Briefly, in a Type-Ⅰ heterojunction (Figure 2a), the CB and VB positions of SC-B are situated entirely within the band gap region of SC-A. This results in a straddling-type band alignment where electrons and holes are confined to SC-B. In a Type-Ⅱ heterojunction (Figure 2b), the CB of SC-B lies below the CB of SC-A, whereas the VB of SC-A is positioned above that of SC-B. This results in a staggered alignment, but electrons and holes are spatially separated. Type-III heterojunction is similar to Type-II, though both the CB and VB of SC-A are positioned higher than those of SC-B, setting the energy bands far apart. This configuration prevents the separation of electron–hole pairs, rendering Type-III heterojunction unsuitable for photocatalytic applications. Hence, the illustration of Type-III is not presented here. A p–n heterojunction (Figure 2c) is similar to a Type-II heterojunction, in which the CB and VB positions of a p-type semiconductor are higher than those of the n-type semiconductor. A Schottky heterojunction (Figure 2d) is formed between a metal and a semiconductor, where a Schottky barrier is created at the junction interface due to their work function difference. For Z-scheme heterojunction (Figure 2e), the photogenerated electrons from the less negative CB edge of SC-B will recombine with the less positive VB edge of SC-A, leaving behind electrons in the CB of SC-A and holes in the VB of SC-B. The band structure of the S-scheme heterojunction (Figure 2f) is similar to that of Type-II (staggered type) heterojunction, but it has a distinct charge transfer route that follows a macroscopic “step” pattern. The S-scheme heterojunction is usually composed of a reduction photocatalyst (SC-A) and an oxidation photocatalyst (SC-B). The SC-A has higher CB and VB positions compared to SC-B.

In a Type-I heterojunction (Figure 2a), the VB and CB of SC-B are positioned within the forbidden band region of SC-A. The CB potential of SC-A is more negative compared to SC-B. Therefore, photogenerated electrons in the CB of SC-A can easily transfer to the CB of SC-B, whereas the holes transfer from the VB of SC-A to the VB of SC-B. Owing to the fast recombination between electrons and holes accumulated in the same semiconductor (SC-B), Type-I heterojunctions are ineffective in improving photocatalytic performance. In a Type-II heterojunction (Figure 2b), which is the most common type, the CB potential of SC-A is relatively more negative, and the VB potential of SC-B is relatively more positive. Upon light irradiation, photogenerated electrons in the CB of SC-A will migrate to the CB of SC-B, while holes in the VB of SC-B move to the VB of SC-A. Due to the IEF formed at the junction interface, the photogenerated electrons and holes are effectively separated. However, this separation comes at the expense of weaker reduction and oxidation potentials, resulting in a decrease in the total redox capacity.

Kumar et al. synthesized a CeVO_4_/Ce_2_S_3_ heterojunction (Type-II) in which charge carriers flow opposite to each other, minimizing carrier recombination [35]. Upon light irradiation, the electrons are excited into the CB of CeVO_4_. On the other hand, the oxidation of TEOA (triethanolamine, a sacrificial reagent) substantially increases electron liberation. Subsequently, the electrons are transferred to the CB of Ce_2_S_3_, where they take part in H_2_ production by reducing H^+^ ions. This band gap alignment and edge potential positions of CeVO_4_ and Ce_2_S_3_ follow Type-II heterostructure through staggered band alignment. The holes in the VB of Ce_2_S_3_ move toward the VB of CeVO_4_ and speed up the oxidation of TEOA. This tailored Type-II heterojunction was responsible for the enhanced photocatalytic activity in CeVO_4_/Ce_2_S_3_. Alharthi’s group fabricated GdVO_4_@g-C_3_N_4_ heterojunction for H_2_ evolution [36]. Through the band gap and Mott–Schottky (M-S) studies, the mechanism for the photocatalytic H_2_ evolution was examined, and the existence of a Type-II heterojunction between g-C_3_N_4_ and GdVO_4_ was determined. The CB potentials of g-C_3_N_4_ and GdVO_4_ were determined to be −1.19 eV and −0.36 eV, respectively, while their VB potentials were 1.54 eV and 2.1 eV. Upon light irradiation, electrons in the VB of g-C_3_N_4_ are excited to its CB and subsequently transferred to the CB of GdVO_4_ across the interface, establishing the Type-II heterojunction. These electrons participate in H_2_O reduction, facilitating H_2_ evolution. The photocatalyst achieved a high H_2_ evolution rate of 16,234 μmol g^−1^ in 4 h.

A p–n heterojunction is an effective structure for facilitating efficient transport and separation of charge carriers, formed at the interface between a p-type and an n-type semiconductor. In a p-type, the Fermi level (E_f_) is positioned near the VB, whereas in an n-type, E_f_ is closer to the CB. When these materials come into contact, the difference in E_f_ levels drives electron migration from the n-type to the p-type semiconductor near the junction interface, creating positively charged species. Simultaneously, holes diffuse from the p-type into the n-type semiconductor, leaving behind negatively charged species. As a result, a p–n heterojunction is established at the situation of Fermi level equilibrium, generating a space charge region with an IEF. When the semiconductors are excited by light, as shown in Figure 2c, the IEF directs photogenerated electrons toward the CB of the n-type semiconductor and holes toward the VB of the p-type semiconductor. Similar to a Type-II heterojunction, the CB and VB positions of a p-type semiconductor are generally higher than those of the n-type semiconductor. However, the p–n heterojunction enables more efficient charge separation due to the combined effects of IEF and band alignment. The formation of a p–n heterojunction can be confirmed by examining the slopes in the M-S plot, where the n-type semiconductor exhibits a positive slope, and the p-type semiconductor shows a negative slope. Several vanadate-based p–n heterojunctions have been explored for photocatalytic H_2_ production. Photocatalytic H_2_ evolution was achieved by Bi_2_O_3_/BiVO_4_ (1–4% doped Bi_2_O_3_) p–n heterojunction under visible light [37]. The highest H_2_ evolution rate was observed for 3% Bi_2_O_3_/BiVO_4_, beyond which efficiency declined due to increased charge–carrier recombination and reduced light absorption. Doping of Bi_2_O_3_ in BiVO_4_ improved the H_2_ production activity.

A Schottky junction (Figure 2d) is created when the semiconductor photocatalyst is combined with a cocatalyst (Pt, Au, Ag, MoS_2_, and graphene). These cocatalysts act as charge carrier traps, facilitating the migration of photogenerated electrons or holes from the semiconductor to the cocatalyst, enabling their spatial separation. The Fermi level difference between the semiconductor and cocatalyst creates a Schottky barrier at the contact interface, encouraging a unidirectional carrier transfer from the photocatalyst to the cocatalyst. For instance, Zn_3_V_2_O_8_ has conduction and valence band potentials of −0.10 and 2.90 eV, respectively. However, the material exhibits low efficiency toward H_2_ production. Hence, to boost the activity of Zn_3_V_2_O_8_, Au can be employed as a cocatalyst due to its superior catalytic and conducting characteristics. The Au metal can enhance charge separation by creating a Schottky barrier across Au and Zn_3_V_2_O_8_ as well as raising the Fermi levels of Zn_3_V_2_O_8_. Additionally, Au broadens the photoresponse range through an inherent SPR effect. Jalil et al. deposited different amounts of Au onto pebble-shaped Zn_3_V_2_O_8_ nanoplates using a chemical reduction method [38]. The bare Zn_3_V_2_O_8_ was synthesized via a co-precipitation process. The effects of band gap, surface area, light intensity, pH, reaction temperature, and sacrificial reagents on the photocatalytic activity were explored. The band gaps of pure Zn_3_V_2_O_8_ and Au@Zn_3_V_2_O_8_ were found as 3.0 eV and 2.9 eV, respectively. Pure Zn_3_V_2_O_8_ showed an absorbance edge at 413 nm, while the absorbance edge extended to 427 nm due to the incorporation of Au into the Zn_3_V_2_O_8_ surface. Au_1.0_@Zn_3_V_2_O_8_ produced an H_2_ amount of 7.05 mmol g^−1^ h^−1^, which was 10-fold greater than that of Zn_3_V_2_O_8_ (0.78 mmol g^−1^ h^−1^), which was attributed to the formation of a Schottky barrier at the contact interface and the SPR-induced electrons. Pt is a more proficient cocatalyst but is limited due to its high cost, which can be solved by reducing the size of Pt particles to the atomic scale. This is a viable strategy due to the advantages of highly dispersed active sites, larger surface area, and improved catalyst usage. Therefore, Ma’s group studied the impact of Pt single-atom cocatalysts on the H_2_ production performance of Ag_3_VO_4_ [39]. Ag_3_VO_4_ can be excited by visible light due to its band gap of 2.20 eV. Theoretically, its CB edge potential (0.04 eV) is more negative than the H^+^ reduction potential, indicating that CB electrons should be capable of reducing protons. However, electron–hole recombination at bulk defect sites may occur before these charge carriers reach the surface for redox reactions. Upon loading Pt single atoms, the electrons in the CB of Ag_3_VO_4_ are transferred to the trap states modified by the Pt atoms, increasing the availability of electrons for H^+^ reduction. Moreover, the charge separation was enhanced, and the interfacial charge transfer resistance was reduced, facilitating the generation of more reactive radicals. The Ag_3_VO_4_/Pt-2 (1.1% Pt) composite achieved an optimal H_2_ evolution of 1400 μmol in 3 h of light irradiation with 20% TEOA (hole scavenger). The activity was sustained for up to four cycles.

As discussed before, while Type-II heterojunction photocatalysts enable spatial separation of photogenerated electron–hole pairs, their redox abilities are weakened, which may hinder their application toward H_2_ evolution due to the high redox potential requirement for the H_2_/H^+^ reaction. In contrast, Z-scheme heterojunctions not only possess strong redox capability for driving photocatalytic reactions but also an effective separation of reductive and oxidative active sites. In direct Z-scheme heterojunctions, photogenerated electrons with a high reduction potential in the CB of SC-A and holes with a high oxidation potential in the VB of SC-B are retained (Figure 2e). When the semiconductors come into close contact, electrons diffuse from SC-A to SC-B, creating a depletion region in SC-A and an accumulation region in SC-B. As a result, opposing charges are created at the interface, causing an IEF to direct from SC-A to SC-B. Moreover, the close contact of two SCs leads to band bending due to the alignment of their Fermi energy levels to the same position. Therefore, IEF, band bending, and coulombic interaction drive the photoexcited electrons in the CB of SC-B to move in the opposite direction to the IEF and make them ultimately recombine with holes in the VB of SC-A at the junction contact. The charge transfer pathway resembles the letter Z, and the charge transfer mechanism retains electrons with high reduction potential in SC-A and holes with high oxidation potential in SC-B. Therefore, direct Z-scheme heterojunction leads to improved separation of charge carriers while maintaining strong redox capacity.

Bismuth vanadate (BiVO_4_) is one of the visible light-responsive photocatalysts that have been widely employed for H_2_ evolution [40]. The transition from Type-II to Z-scheme was realized in CdSe/BiVO_4_ heterojunction by simply altering the order of deposition layers on fluorine-doped tin oxide (FTO) [41]. CdSe has strong visible light absorption and better CB potential for H_2_ evolution but suffers from fast charge recombination and photocorrosion. In contrast, BiVO_4_ lacks the required CB potential for H_2_ production. When a BiVO_4_ layer was introduced on top of CdSe (CdSe/BiVO_4_), in situ Cd-doping occurred in BiVO_4_, modifying the energy band structure and enabling Z-scheme charge transfer. In contrast, when CdSe was deposited on top of BiVO_4_ (BiVO_4_/CdSe), no Cd-doping occurred, and a Type-II heterojunction formed. Due to its enhanced charge separation, CdSe/BiVO_4_ Z-scheme heterojunction achieved an H_2_ amount of 2.59 μmol cm^−2^ h^−1^. Additionally, it exhibited superior stability, maintaining 82% of its initial activity after four cycles, whereas CdSe and BiVO_4_/CdSe degraded due to photocorrosion. The improved performance of CdSe/BiVO_4_ is assigned to the Z-scheme charge transfer, which enhanced electron reduction ability and prevented CdSe from direct oxidation and reaction with active oxygen species. This study demonstrated a facile but efficient strategy to optimize CdSe/BiVO_4_ heterojunctions for efficient and stable photocatalytic H_2_ production. Compared to single *Z*-scheme heterojunctions, dual Z-scheme heterojunctions have higher photocatalytic activity. A heterojunction composite of g-C_3_N_4_/BiVO_4_/CoFe_2_O_4_ was prepared via a high-temperature solid-state reaction by Gong’s group [42]. The photocatalyst exhibited significantly enhanced activity compared to individual components (g-C_3_N_4_, BiVO_4_, and CoFe_2_O_4_) and their binary counterparts.

Conversely, all-solid-state Z-scheme heterojunction consists of a combination of different semiconductors along with metal/non-metallic elements as electron mediators. An all-solid-state g-C_3_N_4_/Au/BiVO_4_ *Z*-scheme photocatalyst was synthesized by integrating gold (Au) nanoparticles at the g-C_3_N_4_/BiVO_4_ interface [43]. The composite exhibited excellent activity for H_2_ evolution and good stability without any cocatalyst. With methanol as a sacrificial agent, the 5-CN/Au/BiVO_4_ sample (0.5 wt.% Au loading) demonstrated the highest H_2_ evolution rate (2986 μmol g^−1^ h^−1^, five cycles), which was 15 times higher than that of g-C_3_N_4_ (199 μmol g^−1^ h^−1^) and 10 times greater than bare BiVO_4_ (297 μmol g^−1^ h^−1^). This enhancement was owing to the anisotropic junction in the composite, which efficiently separated photogenerated charge carriers. By employing Au nanoparticles as an electron shuttle, charge migration and visible light absorption were effectively improved in the g-C_3_N_4_/Au/BiVO_4_ heterojunction.

The S-scheme (step-scheme) heterojunction system, similar to the Z-scheme system, enhances redox abilities while promoting efficient separation and transfer of photogenerated carriers (Figure 2f). The S-scheme system consists of two n-type semiconductors: SC-A, which serves as a reduction photocatalyst (RP) with a larger work function, and SC-B, an oxidation photocatalyst (OP) with a smaller work function. The carrier transfer pathway resembles a “step” or N-shaped pattern, where electrons from the CB of SC-A recombine with holes in the VB of SC-B. This process results in the spatial separation of electrons and holes with stronger reduction and oxidation potentials. Additionally, the Fermi level difference between the two SCs drives electrons to migrate from SC-A to SC-B until the Fermi level equilibrium is reached. This creates an IEF at the junction interface, which serves as the primary driving force for the migration of photogenerated charge carriers. A p-CuAl_2_O_4_/n-YVO_4_ heterojunction photocatalyst was synthesized by a sol–gel technique using block copolymer (nonionic F-108 surfactant) templating [44]. Upon the formation of p–n heterojunction, owing to the Fermi level (E_f_) difference, electrons migrate from the n-type YVO_4_ to the p-type CuAl_2_O_4_ until the Fermi level equilibrium is reached. Under visible light irradiation, both semiconductors generate electron–hole pairs, with charge transfer occurring due to the energy band alignment. The CB and VB of CuAl_2_O_4_ are higher than those of YVO_4_, leading to electron transfer from CuAl_2_O_4_ to YVO_4_. This movement establishes an electric field at the interface, balancing charge diffusion. The band bending facilitates charge separation, promoting electron–hole recombination at specific energy levels while enabling the oxidation of glycerol at the VB of YVO_4_ and the reduction of H^+^ at the CB of CuAl_2_O_4_, which ultimately leads to H_2_ production via the S-scheme mechanism. The 15% CuAl_2_O_4_/YVO_4_ nanocomposite achieved a H_2_ amount of 2994.6 µmol g^−1^ h^−1^ (up to 5 cycles), which was 72.5 times higher than bare YVO_4_ (41.3 µmol g^−1^ h^−1^).

## 3. Vanadates for Photocatalytic H_2_ Production

### 3.1. Magnesium Vanadates

Magnesium vanadate (Mg_3_V_2_O_8_), a member of the vanadate family, is gaining attention in the research community for its potential in photocatalytic applications due to its notable excitation binding energy and low cost. The presence of multiple oxidation states (V^2+^, V^3+^, V^4+^, and V^5+^) enhances its ability to participate in redox reactions, which boosts photocatalytic efficiency and increases the material’s stability during the catalytic process. As a result, Mg_3_V_2_O_8_ has been adopted as an active electrode material for water oxidation under visible light [45]. Alharthi et al. proposed a low-cost Mg_3_V_2_O_8_-rGO nanocomposite for H_2_ evolution and evaluated the photocatalytic properties by varying the sample dose in different solvents (lactic acid, methanol, triethanolamine, and water) [46]. The Mg_3_V_2_O_8_-rGO composite (E_g_ ~2.98 eV) demonstrated a reasonable amount of H_2_ generation (97.45 μmol g^−1^) with good reusability for up to 4 cycles, which was relatively higher than that of pristine Mg_3_V_2_O_8_ (17.45 μmol g^−1^).

### 3.2. Nickel Vanadates

Nickel oxide (NiO) is a versatile material with numerous advantages, including its multivalent nature (Ni^2+^ and Ni^3+^), large surface area, remarkable electrical conductivity, catalytic activity, durability, affordability, and environmental friendliness [47]. Hence, Ni_3_V_2_O_8_ could have the potential to outperform single-transition-metal oxides, such as V_2_O_5_. Ni_3_V_2_O_8_ has been demonstrated in water oxidation, supercapacitors, lithium/sodium-ion batteries, and photovoltaic energy conversion. Orthorhombic Ni_3_V_2_O_8_ is of particular interest due to its remarkable quantum yield, appropriate band gap (2.4 eV), and outstanding photocatalytic performance [48]. Graphitic carbon nitride (g-C_3_N_4_) is a non-metal 2D semiconductor with a reasonable band gap (2.7 eV) and adequate reduction capacity [49]. However, the practical use of g-C_3_N_4_ is restricted by its low specific surface area, quick recombination of electron–hole pairs, poor light absorption capacity, and ineffective electrical conductivity. To address these limitations, Lokanath’s group formed an S-scheme heterojunction (p–n type) by decorating ultra-thin 2D g-C_3_N_4_ nanosheets with 0D Ni_3_V_2_O_8_ QDs via a facile hydrothermal route [50]. For the heterostructure composites, the characteristic tri-s-triazine unit structure and graphitic sheet stacking of g-C_3_N_4_, along with the orthorhombic structure of Ni_3_V_2_O_8_, were preserved. A cubic-like morphology was obtained due to the agglomeration of Ni_3_V_2_O_8_ QDs. The structural analyses confirmed the formation of Ni_3_V_2_O_8_/g-C_3_N_4_ composites with a uniform dispersion of Ni_3_V_2_O_8_ QDs on g-C_3_N_4_ nanosheets. The band gap energies (E_g_) were found to be 2.86 eV for g-C_3_N_4_ and 2.25 eV for Ni_3_V_2_O_8_ QDs. The strong attachment of Ni_3_V_2_O_8_ QDs (particle size ~3–5 nm) with the g-C_3_N_4_ surface increased the carrier separation, redox potential for surface reactions, and abundant active sites within the heterostructure. Moreover, the formation of a depletion layer at the Ni_3_V_2_O_8_/g-C_3_N_4_ interface was verified, leading to the generation of an IEF oriented from g-C_3_N_4_ to Ni_3_V_2_O_8_ QDs. This facilitated charge migration via the S-scheme heterojunction. The optimum heterostructure, Ni_3_V_2_O_8_/g-C_3_N_4_ (50 wt.% of Ni_3_V_2_O_8_), showed the highest H_2_ evolution rate (3380 µmol g^−1^ h^−1^) under low visible light intensity (25 mW cm^−2^), significantly outperforming the bare Ni_3_V_2_O_8_ and g-C_3_N_4_ components.

**Figure 3:**
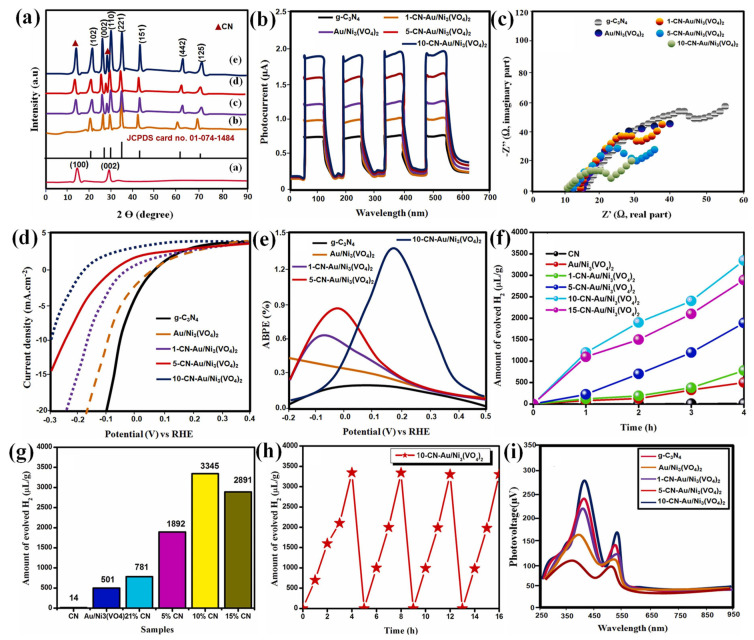
(**a**) PXRD patterns of (**a**) pure CN, (**b**) Au/Ni_3_(VO_4_)_2_, (**c**) 1-CN-Au/Ni_3_(VO_4_)_2_, (**d**) 5-CN-Au/Ni_3_(VO_4_)_2_, and (**e**) 10-CN-Au/Ni_3_(VO_4_)_2_ composites. (**b**) Photoresponse curve, (**c**) EIS plots, (**d**) current density vs. potential plots, (**e**) ABPE curves, (**f**) photocatalytic H_2_ production activity, (**g**) H_2_ evolution rate, (**h**) recyclability test, and (**i**) surface photovoltage curves for the photocatalysts [56]. Copyright 2022, Elsevier.

Noble metal nanoparticles, such as silver (Ag), gold (Au), and platinum (Pt), have garnered tremendous interest because of their intriguing features, which include localized surface plasmon resonance (LSPR) at visible wavelengths, the ability to donate electrons for catalytic reaction, and a high surface-to-volume ratio [51,52,53,54,55]. These metal nanoparticles can work as electron mediators at the junction interface of heterostructure photocatalysts. Muthukumar et al. used Au nanoparticles in a hydrothermally synthesized Z-scheme g-C_3_N_4_-Au/Ni_3_(VO_4_)_2_ (2D/3D) heterostructure to increase visible light absorption [56]. Figure 3a(a–e) depict the PXRD patterns of the pure g-C_3_N_4_ (CN), Au/Ni_3_(VO_4_)_2_, 1-CN-Au/Ni_3_(VO_4_)_2_, 5-CN-Au/Ni_3_(VO_4_)_2_, and 10-CN-Au/Ni_3_(VO_4_)_2_ composites (1, 5, and 10 wt.% of CN). The CN has distinct peaks at ~27.1°(002) and ~13.8°(100). The high crystallinity of Ni_3_(VO_4_)_2_ was indicated by a strong peak at 34.5°. For the CN-Au/Ni_3_(VO_4_)_2_ composite, due to the increasing mass fraction of Ni_3_(VO_4_)_2_, the (002) peak of CN gradually weakened and shifted to longer diffraction angles, indicating the interaction between constituents. The transient photocurrents of samples were examined under light irradiation with 0.3 V (vs. Ag/AgCl) potential in Na_2_SO_4_ solution (Figure 3b). The strongest photoresponse of the composites was ascribed to the rapid generation and transport of charge carriers. Electrochemical impedance spectra (EIS) confirmed the lowest charge transfer resistance (R_ct_) for the CN/Au/Ni_3_(VO_4_)_2_ composite, which was beneficial for better conductivity and faster carrier migration (Figure 3c). As shown in Figure 3d, photocurrent density increased for the composite. The composite with 10 wt.% of CN exhibited the highest photoconversion efficiency (1.35%), credited to enhanced charge transfer between the constituents (Figure 3e). The findings suggest that the absorption of Au nanoparticles at the distinct SPR wavelength (590 nm) influenced the photocatalytic activity. The CN/Au/Ni_3_(VO_4_)_2_ (10 wt%) performed well compared to the pure and other composite samples (Figure 3f,g). For this sample, the Apparent Quantum Efficiency (AQE) was found to be 6.8% at 420 nm. Moreover, H_2_ was continuously produced for 4 h up to five cycles, and this outstanding performance could be due to the rapid separation of photogenerated charge carriers and the surface plasmon resonance (SPR) effect of Au nanoparticles (Figure 3h,i).

### 3.3. Ferric Vanadates

**Figure 4:**
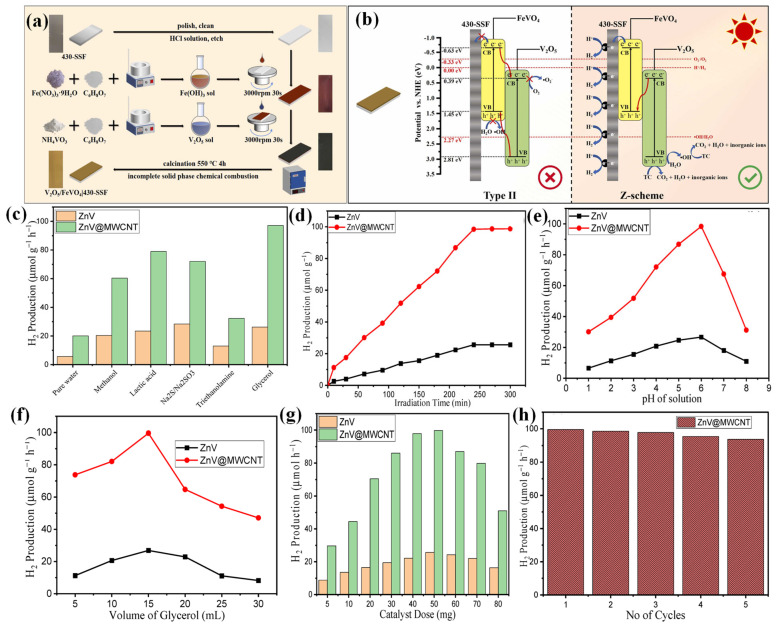
(**a**) Fabrication of V_2_O_5_/FeVO_4_|430-SSF Z-scheme composite film, (**b**) photocatalytic H_2_ production and tetracycline degradation mechanism by the composite [58]. Copyright 2024, Elsevier. Effects of (**c**) various sacrificial reagents, (**d**) irradiation time, (**e**) pH, (**f**) glycerol concentration, and (**g**) catalyst dose on the H_2_ production activity of ZnV and ZnV@MWCNT, (**h**) reusability plot for ZnV@MWCNT [62]. Copyright 2023, Molecules.

The band structure of FeVO_4_ (2.03–2.7 eV) is characterized by a VB primarily consisting of O2p orbitals and a CB dominated by partially filled 3d orbitals, enabling FeVO_4_ to absorb a broad spectrum of visible light [57]. Hence, Tu’s group coupled FeVO_4_ with V_2_O_5_ to prepare the immobilized V_2_O_5_/FeVO_4_|430-SSF composite film, which permitted H_2_ and CO_2_ production separately [58]. As shown in Figure 4a, Fe(OH)_3_ and V_2_O_5_ sols were spin-coated onto 430-stainless steel (SSF), following calcination at 550 °C for 4 h. Due to the incomplete solid-phase chemical reaction, the composite was successively produced, effectively producing H_2_ and CO_2_, as FeVO_4_ and V_2_O_5_ had relatively negative CB and positive CB potentials, respectively. Moreover, as the FeVO_4_’s VB potential was comparatively close to the V_2_O_5_’s CB potential, their combination created a Z-scheme composite (Figure 4b). Since the band gap of FeVO_4_ (2.08 eV) is smaller than that of V_2_O_5_ (2.42 eV), photons with wavelengths longer than 530 nm could penetrate the V_2_O_5_ layer and stimulate the FeVO_4_ layer. Following optimization of various parameters, including the number of coating layers, calcination temperature, and calcination time, the optimum composite film (2 layers of V_2_O_5_, 550 °C, 4 h) produced an H_2_ amount of 370.81 μmol/dm^2^ within 120 min of light irradiation.

### 3.4. Zinc Vanadates

Recently, zinc vanadate (Zn_3_V_2_O_8_) has been paid more attention due to its outstanding optoelectronic properties. Zn_3_V_2_O_8_ (ZnV) has been synthesized in various morphologies, including nanoplatelets, nanospheres, and nanosheets [59,60,61]. The VB of ZnV consists of O2p orbitals, while the CB is dominated by V3d orbitals. Because of its filled 3d orbitals, Zn suffers from strong recombination of charge carriers and possesses a large band gap [57]. Carbon materials, for instance, carbon nanotubes (CNTs), can be a good support for semiconductor photocatalysts due to their unique electronic structure, exceptional surface area, and high electron storage capacity. Particularly, MWCNTs are highly effective owing to their improved electrical conductivity, high aspect ratio, and optical characteristics. Therefore, Alharthi and co-workers combined ZnV with MWCNT [62]. The role of sacrificial reagents (SR) in H_2_ production was investigated. As depicted in Figure 4c, the maximum H_2_ amount of 97.13 μmol g^−1^ h^−1^ was attained for ZnV@MWCNT photocatalyst with glycerol. The irradiation time, solution pH, glycerol concentration, and catalyst dose were optimized (Figure 4d–g). With the increase of irradiation time (0–300 min), the H_2_ evolution rate increased until 240 min and then became constant for both catalysts (Figure 4d). The prolonged time of light irradiation resulted in a high photoabsorption rate, resulting in more generation of •OH/•O_2_^–^ radicals for the water-splitting process. Figure 4e show that with the increase of pH (1–6), the H_2_ production rate also increases and then declines beyond pH 6. At a lower pH value, more H^+^ ions were deviated by the positive surface of the catalyst and thus resulted in a low H_2_ production rate. But, at higher pH levels, the amount of H^+^ ions decreased, leading to a sluggish rate of H_2_ generation. The optimum volume of glycerol was 15 mL (Figure 4f). With the optimized reaction conditions (15 mL glycerol, pH 6, and 4 h light illumination), a maximum H_2_ production efficiency of 99.55 μmol g^−1^ h^−1^ was attained for ZnV@MWCNT (50 mg dose) compared to bare ZnV (26.87 μmol g^−1^ h^−1^) (Figure 4g). As displayed in Figure 4h, ZnV@MWCNT was highly stable without much difference in H_2_ production efficiency after five cycles of use. When a hybrid composite of ZnV/rGO was employed, a decent H_2_ amount of 104.6 μmol g^−1^ was achieved, with good cyclic stability up to 24 h [63]. The Cd_3_V_2_O_8_/ZnV homojunction developed by the same group showed a better H_2_ evolution rate (366.34 μmol g^−1^ h^−1^) and reusability (four cycles) than the individual components in the presence of a methanol–water mixture [57].

### 3.5. Silver Vanadates

AgVO_3_ is considered one of the most attractive and highly efficient visible-light-driven photocatalysts because of its good crystallinity and narrow band gap. The 1D Ag/AgVO_3_ nanowire is an efficient material for stable photocatalytic H_2_ evolution owing to its low band gap (2.4 eV). Furthermore, the Schottky junction created at the junction of Ag/AgVO_3_ could lead to SPR-induced absorption of visible light. However, Ag/AgVO_3_ has a more positive CB potential compared to H^+^ redox potential. To counter this problem, the SnS_2_ (shell) was integrated over the Ag/AgVO_3_ (core) nanostructure to produce high charge carrier efficiency and promote the photogenerated electron–hole pair separation [64]. The core-shell SnS_2_@Ag/AgVO_3_ exhibited six times higher activity with an H_2_ production of 2802 µmol g^−1^ h^−1^ than Ag/AgVO_3_ (435 µmol g^−1^ h^−1^) in lactic acid–water mixture. A AgVO_3_@rGO/Ag_3_PO_4_ (110) heterojunction could efficiently generate H_2_ along with ammonia (NH_3_) during the simultaneous degradation of sulfamethoxazole [65]. The composite exhibited an H_2_ evolution rate of 546.0 μmol g^−1^ in 6 h, which was greater than that of pure AgVO_3_ nanowire (153.6 μmol g^−1^) and Ag_3_PO_4_ (108.7 μmol g^−1^). The high photocorrosion due to the formation of Ag^0^ during light irradiation could be greatly suppressed by the AgVO_3_@rGO/Ag_3_PO_4_ heterojunction, where rGO was an excellent electron acceptor.

**Figure 5:**
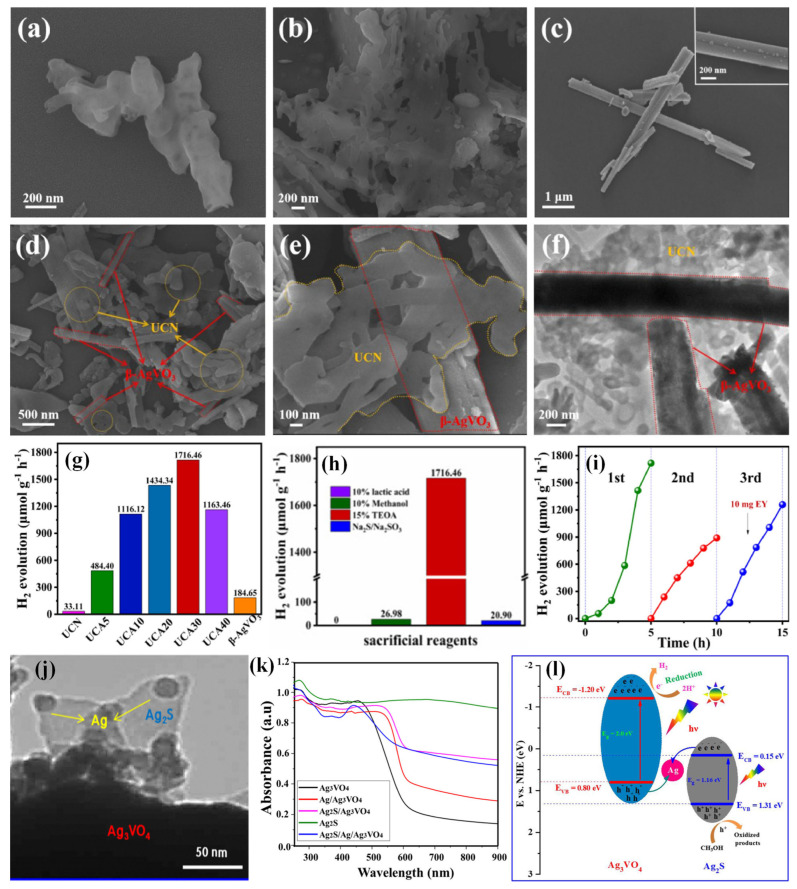
SEM photographs of (**a**) BCN, (**b**) UCN, (**c**) β-AgVO_3_, (**d**,**e**) and UCA30; (**f**) TEM photograph of UCA30; (**g**) H_2_ evolution of UCN, β-AgVO_3_, and UCA*_x_* (*x* = 5, 10, 20, 30, and 40) in 5 h; (**h**) H_2_ generation of UCA30 in various sacrificial reagents; (**i**) cycling test for UCA30 [66]. Copyright 2021, Elsevier. (**j**) TEM image of Ag_2_S/Ag/Ag_3_VO_4_ photocatalyst, (**k**) UV–vis DRS spectra, and (**l**) charge carrier transfer mechanism for all-solid-state Ag_2_S/Ag/Ag_3_VO_4_ Z-scheme photocatalyst [68]. Copyright 2019, Elsevier.

Furthermore, 1D β-AgVO_3_ nanorods were synthesized and integrated with 3D mesoporous ultra-thin g-C_3_N_4_ (UCN) to form porous UCN/β-AgVO_3_ p–n heterojunction (UCA) [66]. Compared to the bulk g-C_3_N_4_ (BCN) shown in Figure 5a, UCN showed abundant pores on its surface, enhancing its specific surface area, active sites, and mass transfer efficiency (Figure 5b). The β-AgVO_3_ displayed nanorod-like morphology (50–200 nm) with some nanoparticles attached to the surface, attributed to reduced silver particles from AgNO_3_ (Figure 5c). As seen in Figure 5d,e, the SEM image of UCA30 shows β-AgVO_3_ nanorods uniformly distributed on porous UCN, which was further confirmed by TEM. The porous UCN layer covered the 1D β-AgVO_3_ nanorods (Figure 5f). The specific surface area of UCA30 (15.02 m^2^/g) was between UCN (38.44 m^2^/g) and β-AgVO_3_ (6.56 m^2^/g), which was reduced due to the presence of β-AgVO_3_ nanorods on the UCN surface. The H_2_ evolution activity was tested using 15% TEOA (triethanolamine, a sacrificial reagent) and EY (eosin Y, a photosensitizer). Due to the synergism between UCN and β-AgVO_3_, UCA30 significantly achieved an H_2_ amount of 1716.46 μmol g^−1^ h^−1^ (Figure 5g). The performance of UCA30 was superior at optimal pH 10 when TEOA (15%) acted as an electron donor and stabilized EY (Figure 5h). Moreover, at lower or higher pH values, the H_2_ production activity was reduced due to variations in reactant behavior and redox potential. The H_2_ evolution peaked at 20 mg of EY because low EY concentrations contributed to limited electron transfer, whereas high EY concentrations caused self-quenching, reducing photocatalytic efficiency. Only UCA30 or EY produced little H_2_, but their combination (UCA30 and EY) showed enhanced activity. The photocatalyst was most active between 400 nm and 450 nm, with the highest H_2_ evolution occurring under 450 nm light. Furthermore, the EY significantly contributed to the stability of UCA30 (Figure 5i). The degradation of EY under prolonged light illumination resulted in a decline in photocatalytic activity. The photocatalytic activity was also affected by the continual use of TEOA (hole scavenger), the protonation of active centers, and imperfect contact in the heterojunction. The UCA30 photocatalyst’s activity was remarkably improved when EY (10 mg) was included again. Hence, the photocatalyst’s stability relied on the EY. The heterojunction system achieved an exceptional H_2_ evolution rate, a 51.8-fold enhancement compared to pure UCN.

Konta et al. first reported the photocatalytic activity of Ag_3_VO_4_, and since then, it has long been employed as a photocatalyst for decomposing hazardous pollutants or generating O_2_ from water [67]. Since Ag_3_VO_4_ has a more negative CB potential than the H^+^ reduction potential, it can be a suitable candidate for H_2_ generation. The hybridization of O 2p, V 3d, and Ag 4d orbitals establishes a narrow band gap, making it a visible-light-sensitive photocatalyst. Loading a cocatalyst is an effective way to increase photocatalytic efficacy via reducing carrier recombination. The cocatalysts can offer active sites for the reduction reaction, trap photoexcited electrons, and reduce the proton reduction activation energy. The photocatalytic activity of Ag_3_VO_4_ was enhanced by Ag nanoparticles through the SPR effect [68]. The hybridization of Ag_3_VO_4_ with Ag nanoparticles provided extended light absorption in the UV–vis range. The morphology of the Ag_2_S/Ag/Ag_3_VO_4_ ternary composite revealed that Ag nanoparticles (20–30 nm size) were grown on the surface of Ag_3_VO_4_ particles and Ag_2_S sheets (Figure 5j). The Ag_2_S exhibited a broad absorption in the visible spectrum. Figure 5k shows a strong absorption edge at 630 nm for Ag/Ag_3_VO_4_, indicating improved visible light absorption. The Schottky junction created between Ag nanoparticles and Ag_3_VO_4_ could facilitate hot electron extraction, improve charge separation efficacy, and encourage overall photocatalytic activity. Since Ag has a larger work function (~4.26 eV), the photogenerated electrons in the CB of Ag_2_S could effectively migrate into Ag nanoparticles via the Schottky junction. Meanwhile, holes in the VB of Ag_3_VO_4_ could easily move into Ag. In this circumstance, Ag acted as an electron mediator or as a recombination center for electrons and holes, contributing to better charge transfer at the junction contact for achieving the complete separation of electrons and holes (Figure 5l). As a result, the Ag_2_S/Ag/Ag_3_VO_4_ composite system produced a high H_2_ amount of 8.5 mmol g^−1^ in aqueous methanol.

More interestingly, in recent years, up-conversion luminescent systems have been employed to increase the solar light utilization capacity of Ag_3_VO_4_. Based on this, a WO_3_:Yb^3+^, Er^3+^/Ag/Ag_3_VO_4_/Ag Z-scheme system was fabricated via a hydrothermal process [69]. The key factors, including irradiation time, SR concentration, and cycle number, were successfully optimized. An absorption edge near 635 nm was revealed by Ag_3_VO_4_, proving that the material could absorb photons in both UV and visible regions. The rare earth (Yb^3+^ and Er^3+^) co-doped WO_3_ nanoparticle acted as a photocatalyst and a luminescent agent to extend photoabsorption. Meanwhile, Ag metal enhanced the electron transfer via the SPR effect and aided as a cocatalyst to boost photocatalytic activity. The nanocomposite achieved H_2_ production of 489.02 μmol g^−1^ in sunlight in 3 h with excellent stability over five cycles.

### 3.6. Indium Vanadates

**Figure 6:**
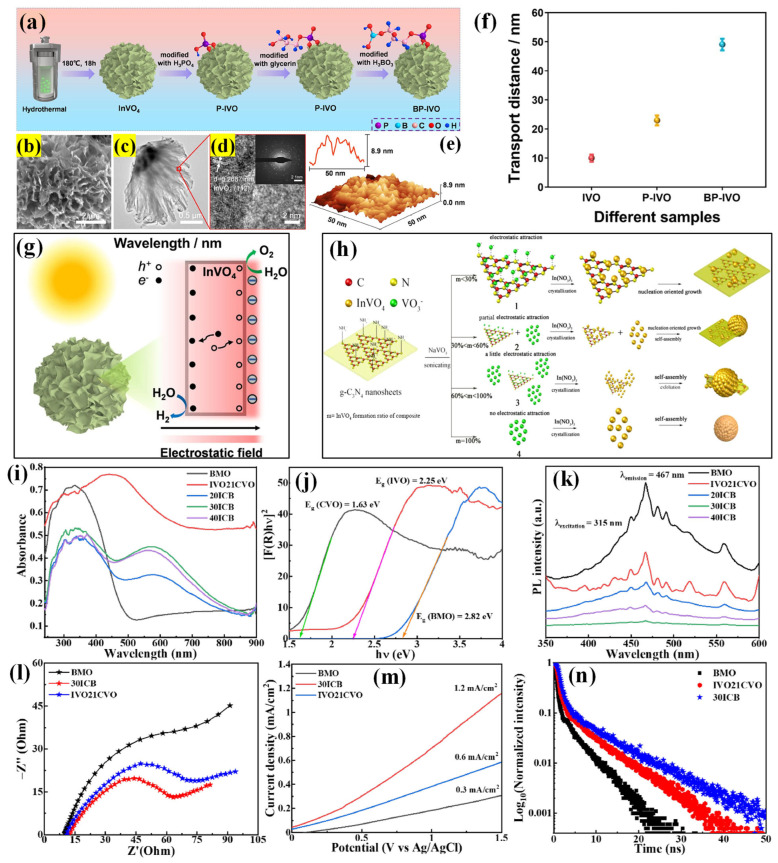
(**a**) Synthesis processes, (**b**,**c**) SEM photographs, (**d**) TEM photograph, (**e**) AFM image of BP-IVO sample, (**f**) hole diffusion length at 350 nm, and (**g**) charge separation and migration under the influence of surface electrostatic field [78]. Copyright 2022, Elsevier. (**h**) g-C_3_N_4_/InVO_4_ composite formation mechanism with various mass ratios of InVO_4_ [80]. Copyright 2015, American Chemical Society. (**i**) UV–vis DRS spectra, (**j**) band gap plots, (**k**) steady-state PL spectra, (**l**) EIS plots, (**m**) LSV plots, and (**n**) TRPL spectra of pure BMO, IVO21CVO binary composite, and 30ICB ternary composite [81]. Copyright 2024, Elsevier.

The visible light activity of InVO_4_ has been attributed to sub-bandgap transitions caused by impurity states. However, recent studies suggest that its actual band-to-band energy gap may exceed 3 eV [70,71,72]. While its extended absorption in the visible spectrum could be beneficial, the presence of impurity states may also lead to unfavorable charge carrier recombination, possibly diminishing the efficacy of InVO_4_ for visible light-driven H_2_ generation. Nevertheless, numerous research groups have explored the significant use of InVO_4_ as a photocatalyst [73,74,75,76,77]. Yu’s group improved hole diffusion length in InVO_4_, a key factor for enhanced water-splitting efficiency, by introducing surface polarization through inorganic acid modification via a wet chemical process [78]. First, hydrothermally prepared InVO_4_ was treated with phosphoric acid (named P-InVO_4_). By using P-InVO_4_ as raw material and glycerol as a “bridge”, the acid (phosphoric and boric acids)-modified InVO_4_ (BP-InVO_4_) was synthesized (Figure 6a). The InVO_4_ possessed a polycrystalline nanoflower-like morphology composed of several nanosheets (Figure 6b–d). The AFM images indicate that the nanosheet possesses a wavy surface, facilitating the connection of inorganic acid groups to it (Figure 6e). They demonstrate that the acid-modified molecule with OH groups ionizes, increasing negative charges on the InVO_4_ surface and creating a negative electrostatic field. This surface polarization directs photogenerated electrons and holes to move in opposite directions, reducing the recombination of charge carriers and extending their diffusion length from 10 nm to 50 nm (Figure 6f,g). The photocatalytic performance was greatly increased, reaching up to 21.74 μmol g^−1^ h^−1^ for H_2_ production and 13.18 μmol g^−1^ h^−1^ for O_2_ production. Since rGO and g-C_3_N_4_ are promising materials to improve the photocatalytic performance of InVO_4_, Neppolian’s group used them with InVO_4_ and demonstrated an excellent H_2_ evolution rate of 7449 μmol g^−1^ h^−1^ [79]. The improved separation and transportation of charge carriers through the rGO layer in the InVO_4_/g-C_3_N_4_ heterojunction resulted in superior H_2_ generation activity.

Shi’s group resolved a fundamental challenge of electron–hole recombination in InVO_4_ in a new way by constructing the heterojunction nanocomposite, which could be extended to several metal oxides and g-C_3_N_4_ [80]. The hydrothermally fabricated g-C_3_N_4_/nano-InVO_4_ composite (80:20 mass ratio) exhibited a remarkable improvement over pure g-C_3_N_4_ or InVO_4_ with an AQE of 4.9% at 420 nm and a notable H_2_ amount of 212 μmol g^−1^ h^−1^. As depicted in Figure 6h, the mechanism of interface formation between InVO_4_ and g-C_3_N_4_ was influenced by the mass fraction of InVO_4_ and g-C_3_N_4_. At lower g-C_3_N_4_ concentrations, VO_3_^3−^ ions could be adsorbed on the positively charged g-C_3_N_4_ sheets through electrostatic attraction, in situ forming InVO_4_ nanocrystals during the reaction process. These nanocrystals further grew into uniformly distributed nanoparticles on the g-C_3_N_4_ sheet surface (process 1). However, as the mass fraction of InVO_4_ increased, the electrostatic attraction weakened, leading to the formation of free InVO_4_ nanoparticles that self-assembled into hierarchical microspheres to reduce interfacial energy (process 2). Further increase in the mass fraction of InVO_4_ nanoparticles led to the exfoliation of g-C_3_N_4_ sheets, forming direct contacts or pure 3D microspheres in the absence of g-C_3_N_4_ (processes 3 and 4). The authors highlighted that the positive charge and 2D structure of g-C_3_N_4_ played a crucial role in this novel approach of synthesizing monodispersed nanojunctions.

In the most recent study, Bi_2_MoO_6_/InVO_4_/CeVO_4_ (BMO/IVO/CVO), a dual S-scheme ternary heterostructure, was fabricated, aiming to improve photocatalytic H_2_ and H_2_O_2_ generation [81]. The work displayed the unique morphological features of the components, such as InVO_4_ nanorods, CeVO_4_ nanosheets, and Bi_2_MoO_6_ nanoplates, fabricated through a simple oil bath-heating process. The ternary heterostructures displayed superior activity compared to their binary analogs and performed well because of the surface oxygen vacancies, which activated atmospheric molecules. The ternary Bi_2_MoO_6_/InVO_4_/CeVO_4_ composite demonstrated an exceptional H_2_ generation rate (2314 μmol g^−1^ h^−1^), surpassing that of the pure components by 12–86 times. Hybridizing BMO with IVO21CVO resulted in the 30ICB ternary composite, which showed enhanced photoabsorption in the UV–vis region (500–800 nm region) (Figure 6i,j). It also helped to mitigate the charge carrier recombination, as verified by PL spectra. The PL feature almost disappeared for the 30ICB composite due to the effective charge transfer (Figure 6k). These separation and migration characteristics were further verified by the EIS plots, revealing the smallest arc radius for the 30ICB composite, demonstrating improved charge carrier mobility (Figure 6l). The LSV curves showed a higher current density (1.2 mA/cm^2^) for the 30ICB (Figure 6m). Moreover, the excited-state lifetime of 30ICB was observed to be 6.5 ns from the time-resolved photoluminescence (TRPL) spectra, attributed to the microscopic junctions-assisted charge migration process (Figure 6n). The extended lifetime of the charge carriers led to the suppression of the recombination process and contributed to the outstanding activity of the composite. The photocatalytic H_2_ production by various vanadate compounds is summarized in Table 1 [82,83,84,85,86,87,88,89,90,91,92,93].

### 3.7. Bismuth Vanadates

Bismuth vanadate (BiVO_4_), with a monoclinic scheelite structure, is a stable and active catalyst in the visible region because of its apt band gap (~2.4 eV) [94,95]. The material consists of Bi 6s, V 3d, and O 2p orbitals in the electronic structure. The presence of Bi 6s orbitals facilitates a shorter migration distance for excited electrons in the VB of BiVO_4_ to the V 3d states in the CB of VO_4_^3−^. This interaction effectively reduces the band gap energy, significantly extending the light absorption range in the visible spectrum [94,96]. These unique properties make BiVO_4_ an exceptional photocatalyst, prompting extensive research on this semiconductor. In past years, BiVO_4_ has been successfully applied in dye degradation [97,98], H_2_ evolution [99,100,101,102], and CO_2_ reduction [103,104]. Nevertheless, its widespread use is restricted by poor electron migration efficiency, sluggish kinetics for oxidation, weak carrier mobility, and poor surface adsorption capacity. Therefore, various strategies have been established to realize the improved photocatalytic activity of BiVO_4_, which include morphology tuning [105], metal doping [106,107], co-doping [108,109], surface engineering [110,111], semiconductor coupling [112,113], deposition of cocatalysts [114,115], use of an electron mediator [116,117], oxygen defect formation (O_V_) [118,119], crystal facet control [120,121,122,123], and heterojunction formation [124,125].

So far, various types of heterojunctions have been developed with BiVO_4_. For instance, BiVO_4_ has been combined with CdS, a reduction photocatalyst, to create a Z-scheme system due to its lower conduction band potential than the H^+^ reduction potential [126,127]. Based on this, CdS (E_g_ ~2.42 eV) and its ternary compounds have been coupled with BiVO_4_ in various morphologies like hollow spheres [128], nanoplates [129], and nanorods [130] with remarkable photocatalytic H_2_ activity owing to their extended photoabsorption capacity, effective charge separation, and photoexcited carrier transport. CdS-decorated BiVO_4_ or BiVO_4_/InVO_4_ artificial leaf Z-scheme heterojunctions have been designed using the biological template, dipping calcination, and SILAR techniques [131,132]. The photocatalyst exposed the highest H_2_ evolution rate of 4188 μmol g^−1^ h^−1^, and the activity declined after three consecutive cycles. Recently, Yang et al. described a hierarchical ZnIn_2_S_4_-NiO/BiVO_4_ Z-scheme heterojunction for effective photodegradation of formaldehyde along with H_2_ generation [133]. The H_2_ amount was 9.082 mmol g^−1^ h^−1^, about 25 times greater than that of ZnIn_2_S_4_. The same group also reported a ZnIn_2_S_4_/BiVO_4_ Z-scheme system decorated with N-C layers, which could act as interfacial channels for rapid charge migration [134]. In this way, ZIS/BVO@N-C-10% unveiled outstanding activity, achieving an H_2_ amount of 2695.68 μmol g^−1^ h^−1^. Similarly, ZnIn_2_S_4_/BiVO_4_/MWCNTs were also reported with a maximum H_2_ amount of 1704 μmol g^−1^ [135]. Additionally, Z-scheme heterojunctions such as BiVO_4_/ZnIn_2_S_4_ [136], BiVO_4_/TiS_2_ [137], Zn_m_In_2_S_3+m_/BiVO_4_ [138], BiVO_4_/Bi_6_O_6_(OH)_3_(NO_3_)_3_ [139], Fe_3_N/BiVO_4_ [140], core-shell BiVO_4_@Fe_2_O_3_ [141], and nickel phthalocyanine/BiVO_4_ [142] have been demonstrated with exceptional visible light absorption, strong stability, effective charge separation and transport, and robust redox capacity.

**Figure 7:**
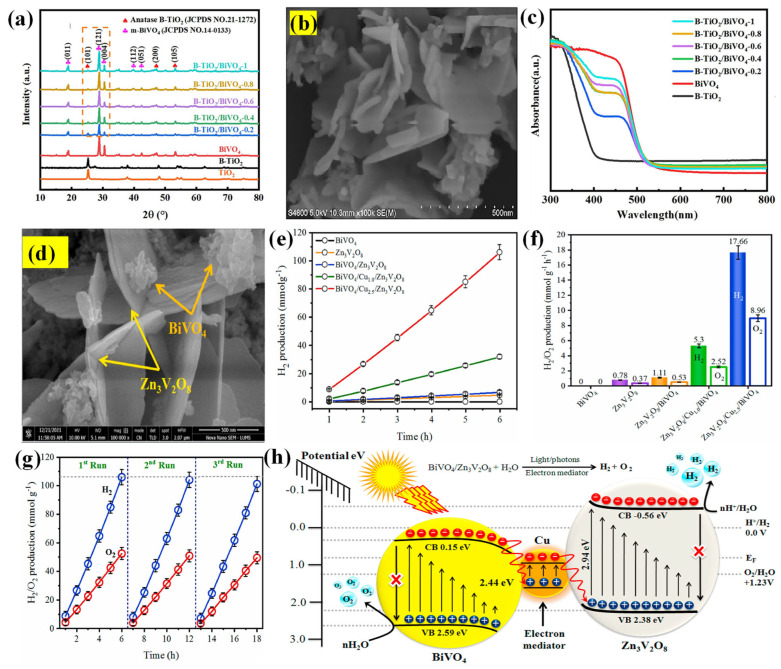
(**a**) XRD of TiO_2_, B-TiO_2_, BiVO_4_, and B-TiO_2_/BiVO_4_-*x*; (**b**) SEM image of B-TiO_2_/BiVO_4_-0.6; and (**c**) UV–vis DRS spectra of B-TiO_2_, BiVO_4_, B-TiO_2_/BiVO_4_-*x* [143]. Copyright 2023, Elsevier. (**d**) SEM image of BiVO_4_/Cu/Zn_3_V_2_O_8_ composite; (**e**) H_2_ evolution rate (in mmol g^−1^); (**f**) H_2_/O_2_ evolution (in mmol g^−1^ h^−1^) of BiVO_4_, Zn_3_V_2_O_8_, BiVO_4_/Zn_3_V_2_O_8_, BiVO_4_/Cu_1.0_/Zn_3_V_2_O_8_, and BiVO_4_/Cu_2.5_/Zn_3_V_2_O_8_ catalysts; (**g**) the recyclability tests for BiVO_4_/Cu_2.5_/Zn_3_V_2_O_8_ catalyst toward H_2_ and O_2_ evolution; and (**h**) Z-scheme mechanism of BiVO_4_/Cu/Zn_3_V_2_O_8_ heterocatalyst [146]. Copyright 2024, Elsevier.

S-scheme heterojunctions of BiVO_4_ can promote the separation of charge carriers and quantum efficiency. Considering its importance, Ma’s group hydrothermally fabricated an S-scheme B-TiO_2_/BiVO_4_ heterojunction system [143]. With BiVO_4_ content increased, the diffraction peak intensity also increased, which confirmed the heterojunction formation (Figure 7a). The B-TiO_2_/BiVO_4_-0.6 exhibited flower-like, ball-shaped B-TiO_2_ nanostructures with smooth, spindle-shaped particles of 50–100 nm in size. As seen in the SEM image (Figure 7b), BiVO_4_ appeared as irregular nanosheets (~50 nm thickness) with granular B-TiO_2_ aggregated on the BiVO_4_ nanosheet surface. The BiVO_4_ served as a support for the formation of B-TiO_2_. Figure 7c depicts the UV–vis DRS of the samples. After incorporating BiVO_4_ into B-TiO_2_, the B-TiO_2_/BiVO_4_ composite revealed strong visible absorption peaks in the 400–550 nm region. The strong contact between BiVO_4_ and B-TiO_2_ encouraged the photogenerated electron–hole separation and suppressed carrier recombination. The H_2_ evolution rate was 561.99 μmol g^−1^ h^−1^ for B-TiO_2_/BiVO_4_-0.6, which was 2.73 times greater than that of B-TiO_2_ (205.62 μmol g^−1^ h^−1^).

Metal oxide composites are promising candidates as photocatalysts due to their capability of creating charge carriers under light illumination, high photoabsorption, excellent charge transport properties, and high excitation lifespan. Various metal oxides have been combined with monoclinic BiVO_4_ [144,145]. The combination of Zn_3_V_2_O_8_ with BiVO_4_ improves conductivity and decreases the overpotential required for water splitting. The alternating Zn─O and V─O layers in Zn_3_V_2_O_8_ lattices afford a favorable structure for interacting with electron mediators. Additionally, the layered architecture of Zn_3_V_2_O_8_ promotes the efficient migration of photogenerated charge carriers to the electron mediator’s surface, thus increasing the photocatalytic activity.

The BiVO_4_/Cu/Zn_3_V_2_O_8_ heterocatalysts were synthesized by varying the amount of Cu(NO_3_)·5H_2_O (0.5–3.5 wt.%) in the mixture of BiVO_4_ and Zn_3_V_2_O_8_ (5 mM each) slurries [146]. The final heterostructure was formed by calcining the mixture at 450 °C for 6 h. Due to the interfacial engineering, the charge transfer became more feasible for redox reactions across the interface. The Cu was in situ added as an electron mediator in the BiVO_4_/Zn_3_V_2_O_8_ heterostructure and played many roles, including (i) preserving synergistic interactions at the interfaces, (ii) facilitating electron transfer, (iii) boosting surface-induced charge generation, (iv) increasing the Fermi energy levels to drive redox reactions, and (v) enhancing the photocatalytic response in sunlight. The authors justified that the band gap reduction (2.65–2.62 eV) was due to the SPR electrons and the charge-mediating role of Cu, which introduced new Fermi energy levels during photoreactions. As seen in Figure 7d, the BiVO_4_ nanostructure grew on the leaf-like surface of 2D Zn_3_V_2_O_8_. Overall, the BiVO_4_/Cu_2.5_/Zn_3_V_2_O_8_ catalyst produced an H_2_ amount of 106 mmol g^−1^ in 6 h (17.66 mmol g^−1^ h^−1^) with an AQE of 9.28% (Figure 7e,f). The BiVO_4_/Cu/Zn_3_V_2_O_8_ heterocatalyst provided a stable photoreaction for 6 h. The stability and reusability were evaluated for the optimum photocatalyst (BiVO_4_/Cu_2.5_/Zn_3_V_2_O_8_). As depicted in Figure 7g, a stable photoreaction was noticed for 6 h. The performance was reduced at the second and third runs (4.5%), mainly due to the catalyst loss and its deposition on the reactor wall, which inhibited the incident light. The authors demonstrated the Z-scheme mechanism of BiVO_4_/Cu/Zn_3_V_2_O_8_ (Figure 7h). The interfacial engineering of heterocatalyst improved the photocatalytic performance, resulting in greater charge transfer. The electrons could easily transfer from the CB of BiVO_4_ to the VB of Zn_3_V_2_O_8_ through the Cu electron mediator, which was facilitated by the interfacial energy bands. The purpose of using Cu as an electron mediator was to maintain the synergy at the BiVO_4_/Zn_3_V_2_O_8_ interface, exchange electrons, boost surface-induced charges, elevate the Fermi levels for the redox process, and enhance the photoresponse in sunlight.

Porous g-C_3_N_4_, with its large surface area and abundant surface functional groups, serves as an excellent platform for coupling with other semiconductors, enabling it as a substrate for constructing heterostructures [147]. Due to this advantage, the Z-scheme g-C_3_N_4_/Au/BiVO_4_ realized the highest H_2_ production of 410.0 μmol g^−1^ h^−1^, which was larger than that of pristine g-C_3_N_4_ (2.2 μmol g^−1^ h^−1^), g-C_3_N_4_/BiVO_4_ (23.3 μmol g^−1^ h^−1^), and Au/g-C_3_N_4_ (244.6 μmol g^−1^ h^−1^). The highest AQE (6.8%) was attained at 420 nm. Moreover, the H_2_ production activity could remain unchanged for up to 50 h with five cycles [148]. The reduced graphene oxide (rGO) was combined with BiVO_4_ to produce ternary heterostructures [149,150,151,152].

### 3.8. Rare Earth Vanadates

The metal orthovanadates generally have monoclinic (monazite type) and tetragonal (zircon type) crystal structures [153,154,155]. Due to the tetragonal structure, CeVO_4_ possesses stabilized Ce^3+^ cations even under oxidizing circumstances. However, the photocatalytic activity of CeVO_4_ is poor in the visible region. Usually, the properties of CeVO_4_ can be altered through morphology control, cation doping, cocatalyst incorporation, surface sensitization, and heterojunction formation [156,157,158]. Hydrothermally synthesized MoS_x_/CeVO_4_ composite (10% CeVO_4_) exhibited the highest H_2_ production (295.24 μmol) within 5 h at pH 9 in the presence of TEOA [159]. The enhanced performance was ascribed to the Z-scheme heterojunction formed between rose-like, 3D-layered MoS_x_ nanoflowers and CeVO_4_, which facilitated the availability of more active sites. However, highly acidic or alkaline environments reduced the performance due to TEOA protonation or H^+^ deficiency. As a dye sensitizer, eosin Y (20 mg) effectively promoted visible light absorption and electron transport. The performance declined after four cycles, primarily due to dye degradation and the depletion of TEOA.

**Figure 8:**
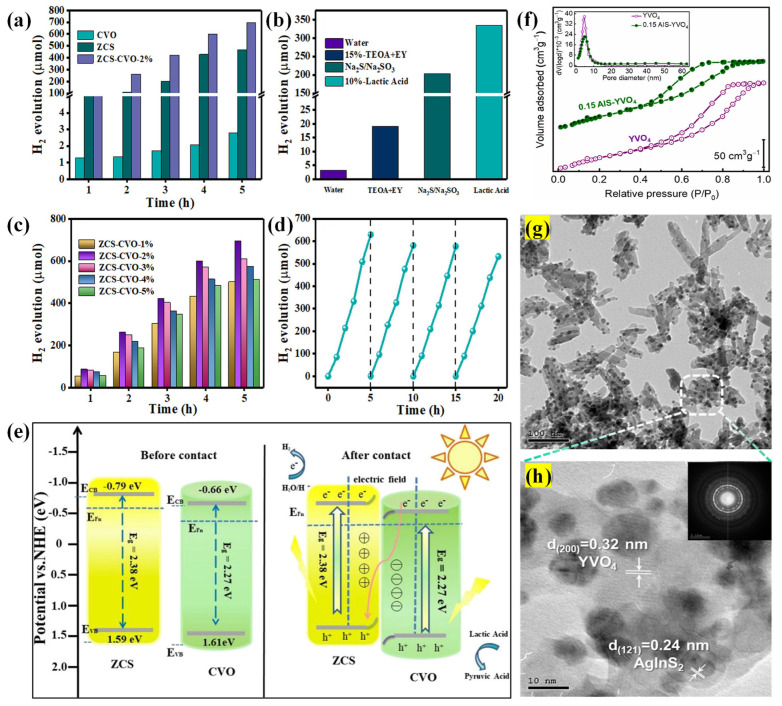
H_2_ evolution rate of (**a**) CVO, ZCS, and ZCS-CVO-2%; (**b**) ZCS-CVO-2% in various sacrificial reagents; (**c**) H_2_ evolution for ZCS-CVO-*x*% (*x* = 1, 2, 3, 4, and 5); (**d**) stability of the ZCS-CVO-2% composite in 20 h; and (**e**) charge transfer in the ZCS-CVO composite system [161]. Copyright 2022, American Chemical Society. (**f**) N_2_ adsorption–desorption isotherms and pore size distribution for YVO_4_ and 0.15 AIS-YVO_4_, (**g**) TEM photograph of 0.15 AIS-YVO_4_, and (**h**) the corresponding lattice spacing measurements and SAED pattern [165]. Copyright 2023, Elsevier.

CeVO_4_ is widely used in catalytic applications due to its unique electrical, optical, and catalytic properties. It offers several advantages, including broad photoabsorption, good crystallinity, and electronic band structure. Due to these attributes, a CoP/CeVO_4_ hybrid photocatalyst was synthesized using a chemical precipitation method [160]. This process involved modifying ZIF-9-derived CoP particles and integrating them with CeVO_4_. The nanohybrid could reach 444.6 μmol of H_2_ in 5 h. Further development in CeVO_4_ was realized in the Zn_0.5_Cd_0.5_S-CeVO_4_ (ZCS-CVO) S-scheme heterojunction fabricated by Zhang’s group [161]. Figure 8a shows the H_2_ evolution activity of the pure and composite samples. The ZCS-CVO (2%) yielded a maximum H_2_ production (695.55 μmol) in a 5 h process with lactic acid (10%). As shown in Figure 8b, ZCS-CVO (2%) exhibited superior activity for lactic acid compared to water, TEOA + EY, and Na_2_S/Na_2_SO_3_. For excessive loading, CVO covered the ZCS host from incident light, resulting in reduced photogenerated charge carriers and, consequently, decreased performance (Figure 8c). After four cycles, each with a 5 h duration, the H_2_ production dropped only by 15.5%, which was due to the lack of lactic acid content (Figure 8d). Based on the above results, the authors proposed a plausible S-scheme mechanism. After bringing ZCS and CVO together to form a close contact, their Fermi levels align due to electron transfer, reaching equilibrium and forming an IEF at the contact interface (Figure 8e). The electron transfer and accumulation cause upward bending of the ZCS band edge and downward bending of the CVO band edge. Due to the synergy of the IEF, energy band bending, and Coulombic forces, visible light irradiation induces free electrons to transfer from the CVO’s CB to the ZCS’s VB. Subsequently, the photogenerated electrons in the ZCS’s CB participate in photocatalytic activity, where H^+^ ions in water are reduced to create H_2_, and holes in the CVO’s VB are oxidized by lactic acid to form pyruvic acid. In another work by Song’s group, CeVO_4_ nanoparticles acted as conductive channels to transfer the photogenerated carriers in the CeO_2_/CeVO_4_/V_2_O_5_ Z-scheme system. CeVO_4_ was created at the junction interface of V_2_O_5_ and CeO_2_ due to the calcination process (450 °C, 550 °C, and 650 °C). The highest H_2_ amount of 189.7 μmol g^−1^ (in 4 h) was observed for the composite calcined at 550 °C for 3 h. Furthermore, CeVO_4_ nanoparticles acted as redox reaction centers to accelerate the electron transfer and effectively separate electrons and holes as well.

Yttrium orthovanadate (YVO_4_), a wide band gap (3.2–3.8 eV) semiconductor, has attracted more attention due to its remarkable photocatalytic features, negative CB potential, and band gap tunability [162,163,164]. YVO_4_ nanorods were hydrothermally combined with AgInS_2_ (AIS, 5–20 wt.%) nanospheres [165]. The adsorption/desorption isotherms revealed that the specific surface area slightly decreased for 15% AIS-YVO_4_ from 170 to 160 m^2^/g (Figure 8f). The optimized composite in the presence of aqueous glycerol and Pt cocatalyst produced a high H_2_ amount of 3.344 mmol g^−1^ h^−1^ with 96% recyclability (five cycles). The controlled incorporation of AIS nanospheres and Pt cocatalyst effectively facilitated the charge distribution and decreased the overpotential for H_2_ production. The TEM image revealed that spherical-shaped AIS nanoparticles (6–12 nm) were attached to the YVO_4_ nanorods’ surface (Figure 8g,h).

The surface of YVO_4_ nanoparticles was in situ decorated by MoS_2_ through a hydrothermal method [166]. In this composite, MoS_2_ acted as an electron trap, effectively suppressing the charge carrier recombination. The 2.5% MoS_2_/YVO_4_ composite revealed the best performance with an H_2_ amount of 134 μmol g^−1^ h^−1^, but YVO_4_ produced only 11.9 μmol g^−1^ h^−1^. The enhanced photocatalytic activity of the MoS_2_/YVO_4_ composite was further boosted by the Pt cocatalyst. The photogenerated electrons accumulated on the Pt nanoparticle surface, reducing H^+^ ions to produce a greater amount of H_2_. In contrast, the 1.0% MoS_2_/YVO_4_ composite exhibited lower activity, and increasing the MoS_2_ amount above 2.5% (up to 12.5%) resulted in a gradual decline in H_2_ evolution. The 2.5% MoS_2_/YVO_4_ composite was stable during the six cycles. The reduced charge carrier recombination and improved performance were attributed to the electron transfer from YVO_4_ to MoS_2_.

Recently, lanthanum vanadate (LaVO_4_) has been recognized as a high-quality photocatalytic material with significant redox potential. But its practical application is hindered by some drawbacks, including high electron–hole recombination (>60%), inferior surface adsorption ability, and sluggish electron migration [167]. The 2D LaVO_4_ nanoflakes, due to their good crystallinity, are promising semiconductors for regulating redox sites through the directional transfer of photogenerated charge carriers. With exceptional catalytic properties and solar light-activated electrons in V3d orbitals, LaVO_4_ has a high H_2_ production efficiency. Using hole scavengers like TEOA or lactic acid is often inefficient and environmentally harmful, as they are oxidized into harmful substances. The SRs can be converted into valuable organic chemicals through the oxidation half-reaction; meanwhile, H_2_ production can be enhanced. Instead of traditional SRs like TEOA, furfuryl alcohol was used with a LaVO_4_/g-C_3_N_4_ (2D/2D) heterostructure by Li and co-workers [168]. This approach not only realized higher H_2_ production but also generated furfural, a valuable byproduct. The TEM image of g-C_3_N_4_ displayed a thin, folded, tulle-like structure, while LaVO_4_ nanoflakes exhibited a uniform square nanosheet structure (~60 nm). The H_2_ yield of the LaVO_4_/g-C_3_N_4_ hybrid composite was much higher than that of pure g-C_3_N_4_ and LaVO_4_. Particularly, the H_2_ evolution rate of 15% LaVO_4_/g-C_3_N_4_ reached 0.287 mmol g^−1^ h^−1^ (AQE ~22.16% at 400 nm), which was 3-fold greater than the pure g-C_3_N_4_. When using TEOA, 15% LaVO_4_/g-C_3_N_4_ demonstrated the greatest H_2_ production of 12.93 mmol g^−1^, which was 9-fold higher than that when furfural was used as the SR. The furfural was less effective than TEOA for H_2_ evolution, but the process could produce more valuable chemicals. The photocatalytic activity of various vanadate compounds for H_2_ production is presented in Table 2 [169,170,171,172,173,174,175,176,177,178,179,180,181,182,183,184,185,186,187,188].

## 4. Conclusions and Future Perspective

This review covers the advancements in metal vanadate-based photocatalysts for H_2_ production under visible light. Numerous design strategies have been reviewed, aiming to improve the performance of metal vanadate photocatalysts, which include metal/non-metal doping, cocatalyst incorporation, nanocarbon loading, vacancy engineering, morphology management, heterojunction construction, and facet engineering. Doping of metal/non-metal into the vanadate lattice could reduce its bandgap and increase light-harvesting capacity in the visible region. Cocatalyst loading plays a crucial role in increasing the light absorption ability via the SPR effect and lowering the recombination loss of photogenerated charge carriers. Combining with nanocarbon could increase light absorption efficiency. The surface vacancies acted as charge trap centers to increase charge carrier separation. Furthermore, the morphology of metal vanadates possesses the strong potential to exhibit higher photocatalytic activity due to a high surface area. Vanadates exhibited excellent redox potentials and effective separation of photogenerated electron–hole pairs, facilitated by Z-scheme/S-scheme charge transfer pathways. The crystal facets play a crucial role in influencing photocatalytic performance by regulating charge carrier dynamics and determining the availability of active reaction sites.

Although some promising results have been achieved, research on metal vanadates still needs further development. Remarkably, existing studies still face issues related to the poor efficiency and stability of vanadate-based materials, which fall short of industrial requirements. Other intrinsic challenges are poor surface area and incomplete formation of heterojunctions at the microscopic level. BiVO_4_, AgVO_3_, and InVO_4_ have been mostly investigated. Recently, rare earth vanadates, particularly YVO_4_ and CeVO_4_, are gaining researchers’ attention for H_2_ production. The modular design of metal vanadate materials simplifies the integration of required functionalities through the proper selection of heterojunction. Properly designed metal vanadates can take advantage of large surface areas, excellent absorption of visible/UV light, and favorable charge transport characteristics. Until now, most of the metal vanadates possess a Type-II or Z-scheme heterojunction system, which has been demonstrated to be outstanding for harvesting visible light and deterring the recombination of photogenerated charge carriers. Nevertheless, only a few studies have focused on the S-scheme/dual Z-scheme heterojunctions for photocatalytic H_2_ evolution. Therefore, exploring more active vanadate-based heterojunction systems would be attractive in the future to achieve spatial separation of photogenerated charge carriers with a longer lifespan of carriers. Moreover, the mechanisms of these heterojunctions remain largely unclear and must be extensively studied.

For a highly efficient metal vanadate photocatalyst, the band gap energy should cover light harvesting in visible and UV regions, while the density of recombination centers should be reduced for effective photocatalytic ability. The diffusion length of photogenerated charge carriers should be greater than the light absorption depth. A rapid rate of surface redox reactions with adequate stability is preferred. In a nutshell, reduced recombination loss of photogenerated electron–hole pairs and enhanced light absorption ability greatly improve photocatalytic performance. As a beacon of guidance for H_2_ production, the physicochemical requirements for the successful development of metal vanadates can be summarized as follows:

✓ Heterojunction formation with metal vanadates, though difficult to obtain, is undoubtedly beneficial for photocatalytic H_2_ production. The heterojunctions can be designed by combining metal vanadates with rare earths, MOFs, and MXenes;
✓ The SPR effect of the noble metal nanoparticles occurring on the surface of metal vanadate can be enhanced;
✓ The crystal facet of metal vanadates can be manipulated to expose more active crystal faces to achieve high photocatalytic performance;
✓ Instead of precious metals such as Pt, Ag, and Au, cost-effective metals such as Cu and Bi can be used as electron mediators;
✓ Chemical stability must be taken into account when developing metal vanadate photocatalysts with a long lifespan, which is a prerequisite for practical applications;
✓ A deeper understanding of the photocatalytic mechanisms of a given metal vanadate can be supported by density functional theory (DFT) calculations and analytical spectroscopic techniques, such as X-ray photoelectron spectroscopy (XPS), X-ray absorption spectroscopy (XAS), and ultrafast transient absorption spectroscopy (TAS).

## Figures and Tables

**Table 1:** Vanadate photocatalysts for H_2_ production.

S. No.	Photocatalyst	Heterojunction Type	Synthesis Method	Light Source	Stability/AQE	Cocatalyst/ Photosensitizer/Sacrificial Agent	H_2_ Evolution	Ref.
1	Mn(bpy)V_4_O_11_(bpy)	---	Hydrothermal	1000 W Xe arc lamp (λ > 420 nm)	---	Pt/Methanol	92 μmol H_2_/^1^/_2_O_2_ g^−1^ h^−1^ (7 h)	[82]
2	Cr-doped Cu_3_V_2_O_8_	---	Hydrothermal	400 W metal halide lamp (λ > 420 nm)	---	NaIO_3_	288 μmol g^−1^ h^−1^	[83]
3	FeVO_4_/CdS	---	Solvothermal	5 W white light (λ > 420 nm)	---	Lactic acid	390 μmol g^−1^ h^−1^(5 h)	[84]
4	rGO/FeVO_4_	---	Hydrothermal	300 W Xe lamp	---	Ethanol	2100.4 μmol g^−1^ h^−1^ (5 h)	[85]
5	Zn_3_V_2_O_8_/Ag	---	Hydrothermal	350 W Xe lamp (λ > 420 nm)	6 cycles	Lactic acid	37.52 μmol g^−1^ h^−1^	[86]
6	AgVO_3_/rGO/CuFe_2_O_4_	---	Hydrothermal	300 W Xe lamp	6 cycles	Lactic acid	8930 μmol g^−1^ h^−1^	[87]
7	Ag/AgVO_3_/g-C_3_N_4_	Z-scheme	Wet impregnation	500 W halogen lamp	3 cycles (9.95%)	---	3570 μmol h^−1^	[88]
8	graphdiyne/β-AgVO_3_	S-scheme	Mechanical ball milling/hydrothermal	300 W LED lamp	3 cycles (10.37%)	EosinY/TEOA	14150 μmol g^−1^ h^−1^	[89]
9	β-AgVO_3_/CdS	p–n	Hydrothermal	300 W Xe lamp	2 cycles (8.02%)	Lactic acid	581.5 μmol (5 h)	[90]
10	AgVO_3_/Pt/g-C_3_N_4_	Z-scheme	Hydrothermal	300 W Xe lamp (λ > 420 nm)	4 cycles (8.1%)	TEOA	10444 μmol g^−1^ h^−1^	[91]
11	MoS_2_/InVO_4_	---	Hydrothermal	300 W Xe lamp (λ > 420 nm)	3 cycles	---	206.4 μmol h^−1^ g^−1^	[92]
12	(NH_4_)_2_V_6_O_16_	---	Hydrothermal	300 W Xe lamp	---	---	140.8 μmol g^−1^	[93]

**Table 2:** Vanadate photocatalysts for H_2_ production.

S. No.	Photocatalyst	Heterojunction Type	Synthesis Method	Light Source	Stability/AQE	Cocatalyst/Photosensitizer/ Sacrificial Agent	H_2_ Evolution	Ref.
1	ZnRh_2_O_4_/Au/ BiVO_4_	Z-scheme	Solid-state reaction/solid–liquid reaction	300 W Xe lamp (λ > 420 nm)	0.056%	---	0.11 μmol h^−1^	[169]
2	BiVO_3_/SnO_2_	S-scheme	Sol–gel/sonochemical	300 W VL Xe lamp (λ > 420 nm)	---	---	8600 μmol g^−1^ h^−1^	[170]
3	BiVO_3_/g-C_3_N_4_	S-scheme	Sonochemical	350W Xe lamp	6 cycles	---	6800 μmol g^−1^ h^−1^	[171]
4	BiVO_4_/polymeric CN	Z-scheme	Hydrothermal	300 W Xe lamp	4.4%	---	14.0 μmol h^−1^	[172]
5	Ti_3_C_2_/BiVO_4_	Schottky junction	Hydrothermal	300 W Xe lamp	---	---	9.39 μmol (4 h)	[173]
6	BiVO_4_/C_3_N_5_	Z-scheme	In situ oil bath	300 W Xe lamp (λ ≥ 420 nm)	0.17%	Pt/Sodium ascorbate	13100 μmol g^−1^ (5 h)	[174]
7	NiFe_2_O_4_/BiVO_4_/Bi_2_MoO_6_	S-scheme	Reflux method	150 W Xe lamp (λ ≥ 420 nm)	4 cycles	TEOA	1110 μmol h^−1^ g^−1^	[175]
8	Ni-MOF-74/BiVO_4_/P	S-scheme	Hydrothermal and calcination	5 W LED (λ ≥ 420 nm)	2 cycles	TEOA	4908 μmol h^−1^ g^−1^	[176]
9	BiVO_4_/Ag/NiFe_2_O_4_	Z-scheme	Hydrothermal	1000 W Xe lamp	---	Na_2_S and Na_2_SO_3_	452 μmol h^−1^ g^−1^	[177]
10	BiVO_4_/Nd-TiO_2_	S-scheme	Electrospinning/hydrothermal	300 W Xe lamp (λ ≥ 400 nm)	---	Methanol	538.44 μmol g^−1^ h^−1^	[178]
11	BiVO_4_/C_3_N_4_-Quinolinic acid	---	In situ solvent evaporation/hydrothermal	300 W Xe lamp (λ > 420 nm)	4 cycles (64.52%)	Pt/TEOA	862.1 μmol h^−1^	[179]
12	Pt-BiVO_4_	---	Precipitation	300 W LED (λ > 420 nm)	4 cycles	Pt/Ethanol	115.7 μmol g^−1^	[180]
13	Bi_0.5_Y_0.5_VO_4_ solid solution	Type-II	Coprecipitation/solid-state reaction	300 W Xe lamp (≥300 nm)	3.25%	Rh@Cr_2_O_3_/ Na_2_S and Na_2_SO_3_	181.3 μmol h^−1^	[181]
14	Pt-CeVO_4_	---	Aqueous phase synthesis	300 W Xe lamp	---	EosinY/TEOA	220680 μmol g^−1^ h^−1^ and 319,780 μmol g^−1^ (18 h)	[182]
15	ZnO/CeVO_4_	Z-scheme	Solvent-assisted reaction/hydrothermal	300 W Xe lamp	5 cycles	Na_2_S and Na_2_SO_3_	1289 μmol h^−1^ g^−1^	[183]
16	AgVO_3_/YVO_4_	---	Sol–gel	500 W Xe lamp (λ > 420 nm)	5 cycles	Pt	3750 μmol g^−1^ h^−1^	[184]
17	GO-C_3_N_4_-LaVO_4_	---	Hydrothermal	300 W Xe lamp	5 cycles	TEOA	717.6 μmol g^−1^ h^−1^	[185]
18	LaVO_4_/g-C_3_N_4_	Z-scheme	Hydrothermal/rotavaporating	300 W Xe lamp (λ ≥ 400 nm)	4 cycles	Pt	890 μmol h^−1^ g^−1^	[186]
19	LaVO_4_/Mo-doped S_V_-ZnIn_2_S_4_	Z-scheme	Hydrothermal/solvothermal	300 W Xe lamp (λ ≥ 420 nm)	4 cycles (30.57%)	TEOA	8670 μmol g^−1^ h^−1^	[187]
20	GdVO_4_	---	Hydrothermal	1000 W Xe lamp (λ > 420 nm)	3 cycles	Methanol	42 μmol h^−1^ (nanowires, UV–vis), 23 μmol (spheres, 4 h visible light)	[188]

## Data Availability

No new data were created or analyzed in this study.

## Abbreviations

AFM	Atomic Force Microscopy
AQE	Apparent Quantum Efficiency
CB	Conduction Band
CNTs	Carbon Nanotubes
COFs	Covalent Organic Frameworks
DFT	Density Functional Theory
DRS	Diffuse Reflectance Spectroscopy
EIS	Electrochemical Impedance Spectroscopy
HRTEM	High-Resolution Transmission Electron Microscopy
IEF	Internal Electric Field
LSPR	Localized Surface Plasmon Resonance
LSV	Linear Sweep Voltammetry
MOFs	Metal-Organic Frameworks
MWCNTs	Multi-walled Carbon Nanotubes
NIR	Near-Infrared
NHE	Normal Hydrogen Electrode
OP	Oxidation Photocatalyst
PL	Photoluminescence
PXRD	Powder X-ray Diffraction
rGO	Reduced Graphene Oxide
RP	Reduction Photocatalyst
SAED	Selected Area Electron diffraction
SC	Semiconductor
SEM	Scanning Electron Microscopy
SPR	Surface Plasmon Resonance
SR	Sacrificial Reagent
TAS	Transient Absorption Spectroscopy
TEM	Transmission Electron Microscopy
TEOA	Triethanolamine
TRPL	Time-Resolved Photoluminescence
UV-VIS	Ultraviolet-Visible
VB	Valence Band
XAS	X-ray Absorption Spectroscopy
XPS	X-ray Photoelectron Spectroscopy
